# Solid subtype of adenoid cystic carcinoma of the breast with multiple distant metastases: a case report and literature review

**DOI:** 10.3389/fonc.2025.1565175

**Published:** 2025-04-22

**Authors:** Yibo Zhang, Xidie Li, Yaxi Xue, Xiaohui Huang, Fengxian An, Miduo Tan

**Affiliations:** ^1^ Department of Ultrasonography, The Hunan Province Directly Affiliated TCM Hospital, Zhuzhou, Hunan, China; ^2^ Department of Obstetrics and Gynecology, Zhuzhou Hospital Affiliated to Xiangya School of Medicine, Central South University, Zhuzhou, Hunan, China; ^3^ Department of Cardiology, Zhuzhou Hospital Affiliated to Xiangya School of Medicine, Central South University, Zhuzhou, Hunan, China; ^4^ Department of Breast Surgery, Zhuzhou Hospital Affiliated to Xiangya School of Medicine, Central South University, Zhuzhou, Hunan, China; ^5^ Department of Pathology, Liaocheng People's Hospital, Liaocheng, Shandong, China

**Keywords:** adenoid cystic carcinoma of the breast, triple-negative breast cancer, distant metastasis, treatment, follow-up

## Abstract

**Objective:**

To present a rare case of adenoid cystic carcinoma of the breast (ACCB), solid subtype, with multiple distant metastases, and to analyze its clinical management and differentiation from typical triple-negative breast cancer (TNBC), highlighting the lack of standardized guidelines for this rare entity and providing insights for future therapeutic strategies.

**Methods:**

A 46-year-old female with ACCB was followed for 9 years, documenting metastatic progression, treatment responses, and survival outcomes. A literature review was conducted to compare ACCB and TNBC in terms of clinicopathological features, immunohistochemical profiles, metastatic patterns, and therapeutic strategies.

**Results:**

The patient exhibited aggressive behavior with metastases to the brain, lungs, liver, and kidneys. Systemic chemotherapy (albumin-bound paclitaxel and capecitabine) combined with radiotherapy stabilized the disease, achieving a 9-year survival with preserved quality of life.

**Conclusion:**

ACCB requires differentiation from TNBC due to its unique biological behavior and favorable prognosis. Breast-conserving surgery with radiotherapy may be preferable for localized disease, while systemic chemotherapy should be considered for metastatic solid subtypes. This case underscores the urgent need for consensus guidelines and further research on molecular profiling to refine therapeutic approaches.

## 1 Introduction

For Adenoid cystic carcinoma of the breast (ACCB) is a rare subtype of invasive breast cancer, with an incidence of approximately 0.92 per million people (1), that accounts for less than 0.1% of all breast cancer cases (2). ACCB primarily occurs in postmenopausal women, although cases in men have also been reported (3, 4). Its histopathological features are similar to those of adenoid cystic carcinoma (ACC) found in other tissues, such as the salivary glands and skin. However, ACCB is typically characterized by local growth and low invasiveness, with rare metastasis to axillary lymph nodes or distant organs (5). Classic ACCB typically exhibits favorable biological behavior, whereas the solid and high-grade transformation types are more prone to local recurrence and distant metastasis (6–8). Therefore, it is crucial to recognize these more aggressive forms of ACCB. ACCB is associated with a favorable prognosis, with 5-year, 10-year, and 15-year survival rates of 98%, 95%, and 91%, respectively (9). Surgery is the mainstay of treatment for ACCB, followed by radiotherapy, while the role of adjuvant chemotherapy remains controversial.

This article presents a case of ACCB with widespread systemic metastasis and shares our experience with its treatment and follow-up. Followed by a review of the previous literature, we discuss the clinical presentation, histological features, immunohistochemical results, treatment strategies, and prognosis of ACCB, aiming to provide a deeper understanding of the disease and offer evidence to guide clinical management. Due to the rarity of ACCB and the lack of consensus guidelines, clinicians often extrapolate treatment strategies from TNBC, risking overtreatment. This case highlights the need for tailored management and underscores the importance of distinguishing ACCB from conventional TNBC.

## 2 Case description

**Figure 1:**
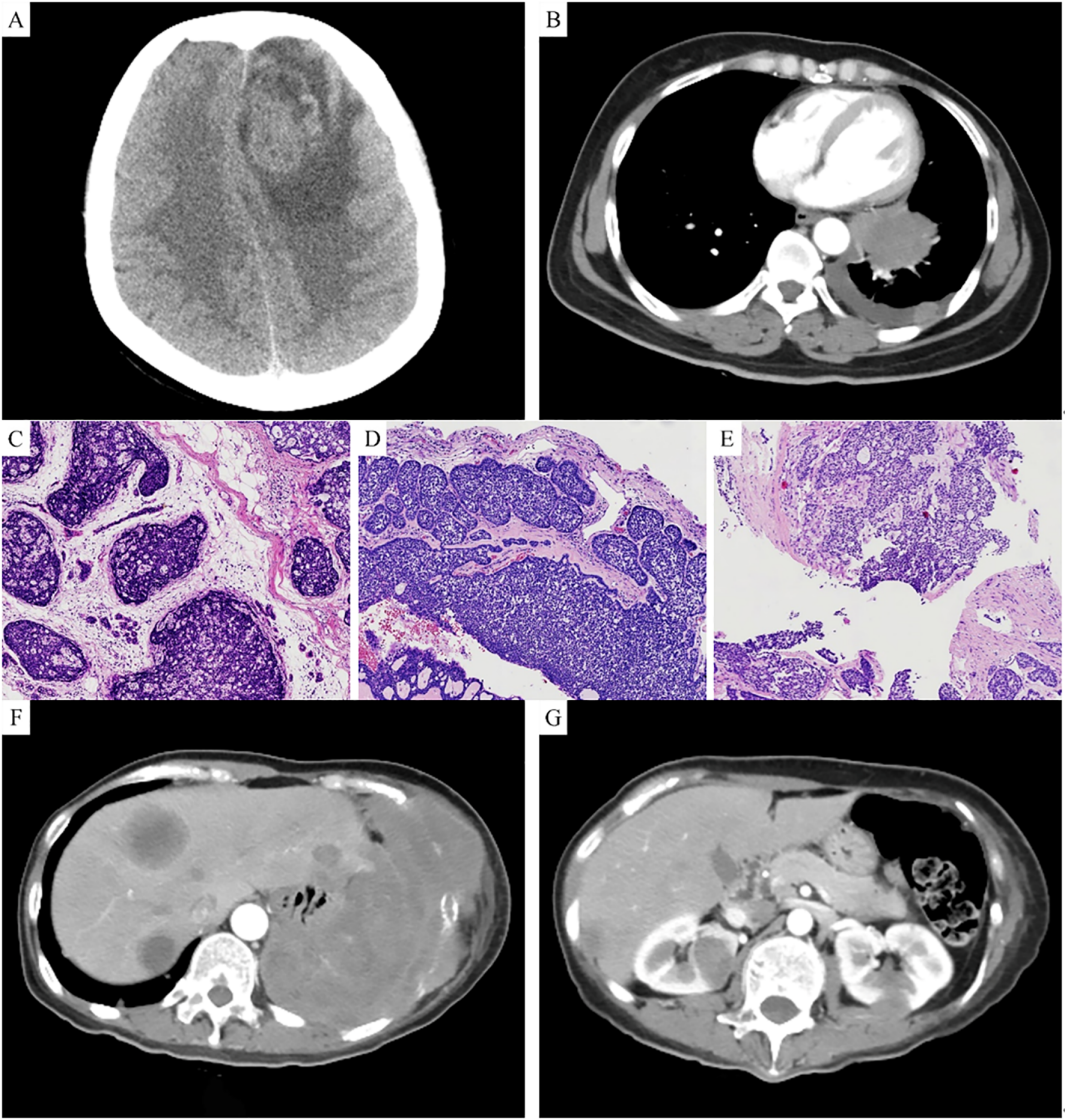
**(A)** MRI shows an intracranial space-occupying lesion. **(B)** CT shows a space-occupying lesion in the left lung. **(C)** Hematoxylin and eosin (HE) staining of the breast tumor shows basaloid cells with deeply stained nuclei. **(D)** HE staining of the brain tumor shows a mixture of basaloid cells and glandular epithelial cells, primarily arranged in a solid pattern. **(E)** HE staining of the lung tumor shows basaloid tumor cells arranged in a solid pattern, with focal sieve-like structures. **(F)** CT shows multiple space-occupying lesions in the liver. **(G)** CT shows multiple space-occupying lesions in both kidneys.

A 46-year-old Han Chinese female, with a BMI of 24.24 kg/m² (height 156 cm, weight 59 kg), was admitted to the hospital in December 2021 due to intermittent headaches for one week. She had no family history of breast cancer and completed high school education. In January 2016, she underwent radical surgery for a breast mass, and the postoperative pathological diagnosis was adenoid cystic carcinoma of the breast (ACCB), solid type (**Figure 1A**). After surgery, she received three cycles of adjuvant chemotherapy with docetaxel (75 mg/m²) and cyclophosphamide (600 mg/m²). Upon admission, relevant examinations were performed. MRI of the head revealed an intracranial mass (**Figure 1B**), and CT of the chest revealed a left lung mass (**Figure 1C**), both of which were suspected to be metastatic tumors.

In December 2021, we resected the left frontal lobe tumor. The histopathological report described the tumor as being composed of two cell types: basaloid cells (nonluminal cells) and glandular epithelial cells (luminal cells). The tumor primarily showed a solid arrangement, with some areas exhibiting tubular, trabecular, and sieve-like patterns of growth. The basaloid tumor cells were round or oval, with scant cytoplasm and prominent mitotic figures (**Figure 1D**). Immunohistochemical profile: ER, PR, HER-2, and GATA3 were all negative, while CD117, P63, CK7, CK, P40, and SOX10 were positive, and Ki67 was 30% positive. Postoperatively, the patient received whole-brain radiotherapy.

In January 2022, we performed a CT-guided biopsy of the left lung mass. Microscopic examination revealed basaloid tumor cells arranged in a solid pattern, with areas of sieve-like structures. The tumor cells had large, deeply stained nuclei with marked atypia and mitotic figures (**Figure 1E**). The immunohistochemical profile was negative for ER, PR, and HER-2 but positive for CD117, P63, CK7, and PAS. On the basis of the histological morphology, immunohistochemical findings, and history of breast cancer, the diagnosis was confirmed as ACCB metastasis to the brain and lungs. After diagnosis, the patient received six cycles of systemic chemotherapy with albumin-bound paclitaxel and capecitabine, with follow-up indicating stable disease.

In February 2024, the patient was readmitted due to right upper abdominal pain. A follow-up CT scan revealed multiple low-density lesions in both the kidney (**Figure 1F**) and the liver (**Figure 1G**), suggestive of metastatic tumors. The patient received ten cycles of systemic chemotherapy with albumin-bound paclitaxel and carboplatin. After completing chemotherapy, the patient was maintained on daily oral capecitabine. As of January 2025, the patient has survived 9 years since the diagnosis of ACCB, with satisfactory quality of life, and is still under follow-up.

## 3 Discussion

ACCB is a rare subtype of TNBC, accounting for approximately 0.1% of all breast cancer cases (2). It predominantly affects postmenopausal women, with a median age of onset between 50 and 60 years (10, 11). ACCB differs from other forms of TNBC in both clinical characteristics and prognosis. Typically, ACCB is characterized by local growth and low invasiveness, with rare metastasis to the axillary lymph nodes or distant organs. The prognosis is generally favorable, with 5-year, 10-year, and 15-year survival rates of 98%, 95%, and 91%, respectively (9).

ACCB often presents as a palpable, solitary breast mass, with a minority of patients experiencing breast pain (12, 13). Symptoms such as nipple inversion, nipple discharge, and skin retraction may also rarely occur (1). The disease typically exhibits slow, indolent growth, with an average diameter of 2–3 cm, although it can reach up to 15 cm (12, 14). A case of giant ACCB with a diameter of 30 cm has been reported, where the mass gradually increased over a span of more than 20 years. Despite the lack of treatment, there are no regional lymph node or distant metastases (15). Although pain is not a typical feature of ACCB, it can serve as an important diagnostic clue (13, 16). Studies have shown that approximately 14% of ACCB patients experience breast pain associated with the mass, possibly due to nerve invasion by tumor cells and the contraction of myoepithelial cells (17). The radiological findings of ACCB lack specificity, and CT scans play an essential role in the follow-up of ACCB patients, as the lungs are the most common site of distant metastasis (16). In the present case, a routine follow-up chest CT revealed a pulmonary mass, and a biopsy confirmed ACCB metastasis to the lungs.

ACCB is primarily composed of glandular epithelial, myoepithelial, and basaloid cells arranged in classic tubular, sieve-like, or solid structures. Invasive growth is commonly observed under a microscope, and some tumors exhibit perineural invasion (12). According to the fifth edition of the WHO classification of breast tumors, ACCB is divided into three histological subtypes: classic, solid, and high-grade transformation. The classic type of ACCB is characterized by glandular epithelial and myoepithelial cells surrounding true and pseudoglandular lumens, with pseudoglandular lumens containing basement membrane-like material. The solid type of ACCB builds upon the classic type, featuring solid cell nests made of basaloid cells, marked cytologic atypia, frequent mitoses, and necrosis. The high-grade transformation type develops further from the classic type and is characterized by high-grade malignant tumor components (4, 18).

ACCB typically does not express ER, PR, or HER-2. Unlike other TNBCs, ACCB displays a unique immunophenotype. Glandular epithelial cells commonly express CK7, CK8, CK18, and CD117, whereas myoepithelial and basaloid cells typically express p63, S-100, CK5, CK6, CK14, and CK17 (4, 18, 19). The expression of p63 and CD117 via immunohistochemistry can help distinguish ACC from invasive cribriform carcinoma and ductal carcinoma in situ (19). Ki-67 expression is relatively low in ACCB (8, 20), with increased expression only in the solid subtype with basaloid cell features, which is consistent with the findings of this study (21). Although ACCB is classified as TNBC, some studies have reported rare cases of hormone receptor-positive ACCB (11).

Surgery is the primary treatment for ACCB. In a single-center study with a follow-up period of 17 years, all ACCB patients underwent surgical treatment (1). Gomez et al. (22) evaluated the benefits of radiotherapy in ACCB patients and reported that postoperative radiotherapy could improve overall survival (OS). Fewer studies have evaluated the value of adjuvant chemotherapy in ACCB. Some studies suggest that ACCB patients do not benefit from adjuvant chemotherapy (23). However, Liu et al. (12) indicated that adjuvant chemotherapy may be beneficial for ACCB patients with distant metastasis. In the present case, the patient underwent radical surgery and adjuvant chemotherapy, which was in accordance with the recommended treatment protocol for TNBC. Further studies with larger sample sizes are needed to better define the treatment strategy for ACCB. Wenig et al. (24) reported two ACCB patients with IDH2 and FGFR2 mutations who were treated with enasidenib and erdafitinib targeted therapies, both of which showed clinical benefit, suggesting that genetic testing plays an important role in the treatment of rare malignant tumors. This case reinforces the importance of differentiating ACCB from conventional TNBC and adopting individualized treatment. It also highlights the potential role of genetic testing in guiding targeted therapies for metastatic ACCB.

Distant metastasis is rare in ACCB, with an incidence of 2.2% (25). Even after metastasis occurs, the disease tends to progress slowly, allowing for long-term follow-up. Classic ACCB generally has a better prognosis than solid and high-grade transformed ACCB. Li et al. (26) found that tumor size, regional lymph node metastasis, histological grade, AJCC stage, and radiotherapy are important prognostic factors for ACCB patients. Additionally, Tang et al. (3) reported nine male ACCB patients, three of whom developed distant metastasis, suggesting that male ACCB patients may have more aggressive behavior and a greater tendency for distant metastasis.

## 4 Conclusion

In conclusion, ACCB is a distinct subtype of TNBC with a favorable prognosis, and its clinical characteristics differ from those of other pathological types of TNBC. Therefore, treatment strategies should be differentiated from those used for TNBC. Clinicians must recognize and distinguish ACCB from other types of TNBC to avoid misclassifying and treating ACCB as TNBC. Breast-conserving surgery followed by postoperative radiotherapy may be a suitable treatment option for ACCB. This case highlights the necessity of genetic profiling in metastatic ACCB. Recent studies identified IDH2 and FGFR2 mutations in ACCB, suggesting potential targeted therapies such as enasidenib or erdafitinib. Collaborative efforts through platforms like the Adenoid Cystic Carcinoma Research Foundation (ACCRF) are critical to accelerate clinical trials for rare cancers. Regular imaging follow-up is essential to exclude distant metastasis. Our findings support the use of breast-conserving surgery combined with radiotherapy for localized ACCB, as it achieves comparable survival to mastectomy while preserving quality of life. However, for solid subtypes with high Ki-67 (e.g., 30% in this case), adjuvant chemotherapy may be warranted despite limited evidence. Furthermore, larger sample studies are needed to identify the most appropriate treatment strategies for ACCB.

## Data Availability

The original contributions presented in the study are included in the article/supplementary material. Further inquiries can be directed to the corresponding authors.
